# Epidemiology of Cryptosporidiosis, New York City, New York, USA, 1995–2018^1^

**DOI:** 10.3201/eid2603.190785

**Published:** 2020-03

**Authors:** Lisa Alleyne, Robert Fitzhenry, Kimberly A. Mergen, Noel Espina, Erlinda Amoroso, Daniel Cimini, Sharon Balter, Ana Maria Fireteanu, Anne Seeley, Lorraine Janus, Bruce Gutelius, Susan Madison-Antenucci, Corinne N. Thompson

**Affiliations:** New York City Department Health and Mental Hygiene, Long Island City, New York, USA (L. Alleyne, R. Fitzhenry, E. Amoroso, D. Cimini, S. Balter, A.M. Fireteanu, B. Gutelius, C.N. Thompson);; New York State Department of Health, Albany, New York, USA (K.A. Mergen, N. Espina, S. Madison-Antenucci);; New York City Department of Environmental Protection, Corona, New York, USA (A. Seeley, L. Janus)

**Keywords:** cryptosporidiosis, Cryptosporidium spp., parasites, epidemiology, sexual and gender minorities, men who have sex with men, travel, international travelers, syndromic multiplex panels, enteric infections, New York City, New York, United States

## Abstract

Cryptosporidiosis has increased and occurs in men who have sex with men and international travelers.

## Medscape CME ACTIVITY

In support of improving patient care, this activity has been planned and implemented by Medscape, LLC and Emerging Infectious Diseases. Medscape, LLC is jointly accredited by the Accreditation Council for Continuing Medical Education (ACCME), the Accreditation Council for Pharmacy Education (ACPE), and the American Nurses Credentialing Center (ANCC), to provide continuing education for the healthcare team.

Medscape, LLC designates this Journal-based CME activity for a maximum of 1.00 ***AMA PRA Category 1 Credit(s)*™**. Physicians should claim only the credit commensurate with the extent of their participation in the activity.

Successful completion of this CME activity, which includes participation in the evaluation component, enables the participant to earn up to 1.0 MOC points in the American Board of Internal Medicine's (ABIM) Maintenance of Certification (MOC) program. Participants will earn MOC points equivalent to the amount of CME credits claimed for the activity. It is the CME activity provider's responsibility to submit participant completion information to ACCME for the purpose of granting ABIM MOC credit.

All other clinicians completing this activity will be issued a certificate of participation. To participate in this journal CME activity: (1) review the learning objectives and author disclosures; (2) study the education content; (3) take the post-test with a 75% minimum passing score and complete the evaluation at http://www.medscape.org/journal/eid; and (4) view/print certificate. 

### Release date: February 20, 2020; Expiration date: February 20, 2021

### Learning Objectives

Upon completion of this activity, participants will be able to:

- Analyze the clinical effect and treatment of cryptosporidiosis
- Distinguish the trends in the prevalence of cryptosporidiosis over time in New York, New York
- Assess the effect of cryptosporidiosis on specific populations
- Evaluate the effect of foreign travel and new diagnostic tools on the prevalence of cryptosporidiosis

### CME Editor

**Thomas J. Gryczan, MS,** Technical Writer/Editor, Emerging Infectious Diseases. *Disclosure: Thomas J. Gryczan, MS, has disclosed no relevant financial relationships.*

### CME Author

**Charles P. Vega, MD**, Health Sciences Clinical Professor of Family Medicine, University of California, Irvine School of Medicine. *Disclosures: Charles P. Vega, MD, has disclosed the following relevant financial relationships: served as an advisor or consultant for GlaxoSmithKline; Johnson & Johnson Pharmaceutical Research & Development, L.L.C.; served as a speaker or a member of a speakers bureau for Genentech, Inc.; GlaxoSmithKline.*

### Authors

*Disclosures:* ***Lisa Alleyne, MPA; Robert Fitzhenry, PhD; Kimberly Mergen, MS; Noel Espina, PhD; Erlinda Amoroso, MBBS; Daniel Cimini, MPH; Sharon Balter, MD, MPH; Ana Maria Fireteanu, MPH; Anne Seeley, MPH; Lorraine L. Janus, PhD; Bruce Gutelius, MD;*** *and* ***Corinne Thompson, PhD,*** *have disclosed no relevant financial relationships.* ***Susan Madison-Antenucci, PhD,*** *has disclosed the following relevant financial relationships: received grants for clinical research from Grifols.*
