# Supplementary material for: Epidemiology of Cryptosporidiosis, New York City, New York, USA, 1995–2018
Source: Emerg Infect Dis. 2020 Mar;26(3):409–19. doi: 10.3201/eid2603.190785 (PMC7045836; doi:10.3201/eid2603.190785)
Supplement: Appendix — Additional information on epidemiology of cryptosporidiosis, New York City, New York, USA, 1995–2018. [file 19-0785-Techapp-s1.pdf]

Article DOI: https://doi/org/10.3201/eid2603.190785

# Epidemiology of Cryptosporidiosis, New York City, New York, USA, 1995–2018

## Appendix

**Appendix Table 1.** Characteristics for patients who were interviewed and patients who were not interviewed regarding cryptosporidiosis, New York City, New York, USA, 1995–2018*

| Characteristic | Interviewed, no. (%) | Not interviewed, no. (%) | p value* |
|---|---|---|---|
| Total | 3,295 (82.7) | 689 (17.3) | |
| Age group, y | | | |
| <5 | 313 (9.5) | 26 (3.8) | <0.001 |
| 5–9 | 172 (5.2) | 11 (1.6) | |
| 10–19 | 203 (6.2) | 24 (3.5) | |
| 20–44 | 1,806 (54.8) | 434 (63.0) | |
| 45–59 | 540 (16.4) | 147 (21.3) | |
| >59 | 260 (7.9) | 45 (6.5) | |
| Sex | | | |
| M | 2,183 (66.3) | 531 (77.1) | <0.001 |
| F | 1,112 (33.7) | 157 (22.8) | |
| Sex and age group, y | | | |
| All <20 | 688 (20.9) | 61 (8.9) | <0.001 |
| Men 20–59 | 1,679 (51.0) | 465 (67.5) | |
| Men >59 | 141 (4.3) | 27 (3.9) | |
| Women >20 | 786 (23.9) | 133 (19.3) | |
| Borough | | | |
| Bronx | 591 (17.9) | 121 (17.6) | 0.52 |
| Brooklyn | 670 (20.3) | 158 (22.9) | |
| Manhattan | 1,572 (47.7) | 322 (46.7) | |
| Queens | 406 (12.3) | 77 (11.2) | |
| Staten Island | 51 (1.5) | 8 (1.2) | |
| Race/ethnicity | | | |
| Hispanic | 1,010 (30.7) | 153 (22.2) | <0.001 |
| Non-Hispanic white | 1,234 (37.5) | 143 (20.8) | |
| Non-Hispanic black/African American | 737 (22.4) | 240 (34.8) | |
| Other | 200 (6.1) | 38 (5.5) | |
| Immune status | | | |
| Non-HIV/AIDS | 1,711 (51.9) | 182 (26.4) | <0.001 |
| HIV/AIDS | 1,579 (47.9) | 474 (68.8) | |
| Neighborhood poverty† | | | |
| Low | 938 (28.5) | 148 (21.5) | 0.001 |
| Medium | 805 (24.4) | 161 (23.4) | |
| High | 593 (18.0) | 144 (20.9) | |
| Very high | 889 (27.0) | 213 (30.9) | |

*By $\chi^2$ test.
†Classified by percentage of census tract residents living below the federal poverty line (low, <10%; medium, 10%–<20%; high, 20%–<30%; very high, ≥30%).

**Appendix Table 2.** Characteristics for cryptosporidiosis patients over time, by demographic characteristics, given a diagnosis during 2000–2018 and reported to the New York City Department of Health and Mental Hygiene*

| | Year(s) of diagnosis, no. (%) | | | | |
|---|---|---|---|---|---|
| Characteristic | 2000–2004 | 2005–2009 | 2010–2014 | 2015–2018 | Total |
| No. cases | 704 | 595 | 500 | 740 | 2,539 |
| Age group, y | | | | | |
| <5 | 62 (8.8) | 61 (10.3) | 49 (9.8) | 78 (10.5) | 250 (9.8) |
| 5–9 | 38 (5.4) | 41 (6.9) | 19 (3.8) | 34 (4.6) | 132 (5.2) |
| 10–19 | 46 (6.5) | 44 (7.4) | 26 (5.2) | 57 (7.7) | 173 (6.8) |
| 20–44 | 407 (57.8) | 300 (50.4) | 282 (56.4) | 413 (55.8) | 1,402 (55.2) |
| 45–59 | 123 (17.5) | 109 (18.3) | 99 (19.8) | 107 (14.5) | 438 (17.3) |
| >59 | 28 (4.0) | 40 (6.7) | 25 (5.0) | 51 (6.9) | 144 (5.7) |
| Sex | | | | | |
| M | 511 (72.6) | 403 (67.7) | 352 (70.4) | 484 (65.4) | 1,750 (68.9) |
| F | 193 (27.4) | 192 (32.3) | 148 (29.6) | 255 (34.5) | 788 (31.0) |
| Sex and age group, y | | | | | |
| All <20 | 146 (20.7) | 146 (24.5) | 94 (18.8) | 169 (22.8) | 555 (21.9) |
| Men 20–59 | 418 (59.4) | 302 (50.8) | 284 (56.8) | 355 (48.0) | 1,359 (53.5) |
| Men >59 | 15 (2.1) | 22 (3.7) | 17 (3.4) | 33 (4.5) | 87 (3.4) |
| Women >20 | 125 (17.8) | 125 (21.0) | 105 (21.0) | 182 (24.6) | 537 (21.2) |
| Borough | | | | | |
| Bronx | 121 (17.2) | 135 (22.7) | 98 (19.6) | 129 (17.4) | 483 (19.0) |
| Brooklyn | 151 (21.4) | 142 (23.9) | 120 (24.0) | 186 (25.1) | 599 (23.6) |
| Manhattan | 327 (46.4) | 245 (41.2) | 218 (43.6) | 314 (42.4) | 1,104 (43.5) |
| Queens | 80 (11.4) | 67 (11.3) | 57 (11.4) | 103 (13.9) | 307 (12.1) |
| Staten Island | 25 (3.6) | 6 (1.0) | 7 (1.4) | 8 (1.1) | 46 (1.8) |
| Race/ethnicity | | | | | |
| Hispanic | 212 (30.1) | 179 (30.1) | 135 (27.0) | 194 (26.2) | 720 (28.4) |
| Non-Hispanic white | 224 (31.8) | 150 (25.2) | 155 (31.0) | 310 (41.9) | 839 (33.0) |
| Non-Hispanic black/African American | 206 (29.3) | 195 (32.8) | 132 (26.4) | 122 (16.5) | 655 (25.8) |
| Other/unknown | 62 (8.8) | 71 (11.9) | 78 (15.6) | 114 (15.4) | 325 (12.8) |
| Immune status | | | | | |
| Non-HIV/AIDS | 281 (39.9) | 313 (52.6) | 238 (47.6) | 544 (73.5) | 1,376 (54.2) |
| HIV/AIDS | 420 (59.7) | 276 (46.4) | 254 (50.8) | 190 (25.7) | 1,140 (44.9) |
| Neighborhood poverty* | | | | | |
| Low | 159 (22.6) | 143 (24.0) | 121 (24.2) | 203 (27.4) | 626 (24.7) |
| Medium | 158 (22.4) | 145 (24.4) | 141 (28.2) | 197 (26.6) | 641 (25.2) |
| High | 148 (21.0) | 134 (22.5) | 102 (20.4) | 151 (20.4) | 535 (21.1) |
| Very high | 217 (30.8) | 159 (26.7) | 133 (26.6) | 188 (25.4) | 697 (27.5) |

*Classified by percentage of census tract residents living below the federal poverty line (low, <10%; medium, 10%–<20%; high, 20%–<30%; very high, ≥30%).

**Appendix Table 3.** Comparison of incidence of cryptosporidiosis by demographic groups between 2000–2014 and 2015–2018, New York City, New York, USA*

| | 2000–2014 | | 2015–2018 | | Between year groups |
|---|---|---|---|---|---|
| Characteristic | No. | Median annual incidence/100,000 persons | No. | Median annual incidence/100,000 persons | IRR (95% CI) |
| No. cases | 3,244 | 1.46 | 740 | 2.07 | 1.49 (1.17–1.91) |
| Age group, y† | | | | | |
| <5 | 261 | 2.30 | 78 | 3.10 | 1.65 (1.16–2.35) |
| 5–9 | 149 | 1.26 | 34 | 1.74 | 1.31 (0.79–2.17) |
| 10–19 | 170 | 0.75 | 57 | 1.40 | 2.07 (1.35–3.19) |
| 20–44 | 1,827 | 2.17 | 413 | 3.05 | 1.56 (1.21–2.03) |
| 45–59 | 580 | 1.41 | 107 | 1.68 | 1.16 (0.86–1.56) |
| >59 | 254 | 0.39 | 51 | 0.68 | 1.67 (1.04–2.68) |
| Sex | | | | | |
| M | 2,230 | 2.00 | 484 | 2.66 | 1.42 (1.07–1.89) |
| F | 1,014 | 0.80 | 255 | 1.57 | 1.63 (1.31–2.02) |
| Sex and age group, y† | | | | | |
| All <20 | 580 | 1.26 | 169 | 2.17 | 1.73 (1.27–2.36) |
| Men 20–59 | 1789 | 2.96 | 355 | 3.47 | 1.29 (0.97–1.73) |
| Men >59 | 135 | 0.60 | 33 | 1.05 | 1.82 (1.18–2.80) |
| Women >20 | 737 | 0.74 | 182 | 1.40 | 1.82 (1.30–2.56) |
| Borough of residence | | | | | |
| Bronx | 583 | 1.59 | 129 | 2.04 | 1.24 (0.98–1.58) |
| Brooklyn | 642 | 1.11 | 186 | 1.46 | 1.55 (1.15–2.09) |
| Manhattan | 1,580 | 3.18 | 314 | 4.81 | 1.31 (1.01–1.70) |
| Queens | 380 | 0.60 | 103 | 1.02 | 1.64 (1.29–2.08) |
| Staten Island | 51 | 0.46 | 8 | 0.42 | 0.71 (0.31–1.60) |
| Race/ethnicity‡ | | | | | |
| Hispanic | 969 | 1.45 | 194 | 1.91 | 1.26 (1.04–1.52) |
| Non-Hispanic white | 1,067 | 1.17 | 310 | 2.44 | 1.91 (1.46–2.50) |
| Non-Hispanic black/African American | 855 | 1.77 | 122 | 1.51 | 0.87 (0.60–1.24) |
| Other | 161 | 0.67 | 77 | 1.34 | 1.76 (1.32–2.35) |
| Immune status†‡ | | | | | |
| Non-HIV/AIDS | 1,349 | 0.65 | 544 | 1.53 | 2.37 (1.75–3.22) |
| HIV/AIDS | 1,863 | 55.6 | 190 | 39.04 | 0.62 (0.43–0.91) |
| Neighborhood poverty†§ | | | | | |
| Low | 883 | 1.18 | 203 | 2.18 | 1.65 (1.25–2.18) |
| Medium | 769 | 1.56 | 197 | 1.96 | 1.36 (1.06–1.76) |
| High | 583 | 1.46 | 151 | 2.14 | 1.32 (1.02–1.70) |
| Very high | 917 | 1.87 | 188 | 2.17 | 1.40 (1.09–1.80) |

*ACS, American Community Survey; IRR, incidence rate ratio.

†Incidence and IRR not age-adjusted.

‡All categories had <10 missing values with the exception of race/ethnicity during 2000–2014 (n = 192) and 2015–2018 (n = 37), immune status during 2000–2014 (n = 32), and poverty during 2000–2014 (n = 92).

§Classified by percentage of census tract residents living below the federal poverty line (low, <10%; medium, 10%–<20%; high, 20%–<30%; very high, >30%); population denominator data were as follows for each listed year: 2000–2004 (2000 US Census); 2005–2009 (ACS 2007–2011); 2010 (2010 Census); 2011 (ACS 2009–2013); 2012 (ACS 2010–2014); 2013 (ACS 2011–2015); 2014–2018 (ACS 2012–2016).
