# Supplementary material for: Epidemiology of Cryptosporidiosis, New York City, New York, USA, 1995–2018
Source: Emerg Infect Dis. 2020 Mar;26(3):409–19. doi: 10.3201/eid2603.190785 (PMC7045836; doi:10.3201/eid2603.190785)
Supplement: Related texts

# Review article

## Abstract

Cryptosporidiosis is a parasitic diarrheal infection that is transmitted by the fecal–oral route. We assessed trends in incidence and demographic characteristics for the 3,984 cases diagnosed during 1995–2018 in New York City, New York, USA, and reported to the New York City Department of Health and Mental Hygiene. Reported cryptosporidiosis incidence decreased with HIV/AIDS treatment rollout in the mid-1990s, but the introduction of syndromic multiplex diagnostic panels in 2015 led to a major increase in incidence and to a shift in the demographic profile of reported patients. Incidence was highest among men 20–59 years of age, who consistently represented most (54%) reported patients. In addition, 30% of interviewed patients reported recent international travel. The burden of cryptosporidiosis in New York City is probably highest among men who have sex with men. Prevention messaging is warranted for men who have sex with men and their healthcare providers, as well as for international travelers.

Cryptosporidiosis is marked by watery diarrhea and caused by parasites of the genus *Cryptosporidium*, which are transmitted by the fecal–oral route (*1*). Known risk factors in the United States include recreational water use, contact with animals, sexual activity in which there might be contact with stool, attendance or employment at child care settings, and consumption of contaminated food and water (*1*–*4*). Infection can cause self-limited diarrhea, although treatment is available and recommended for immunocompetent patients with severe or prolonged symptoms (*5*).

Cryptosporidiosis has been a reportable disease in New York City (NYC), New York, USA, since mid-1994 (*6*), and electronic reporting by laboratories has been required since 2006. As has been seen in other jurisdictions in the United States (*7*), the reported incidence of cryptosporidiosis in NYC has been influenced by the recent introduction of syndromic multiplex diagnostic panels, such as BioFire (https://www.biofiredx.com) and Luminex (https://www.luminexcorp.com). These panels enable physicians to screen for a large number of gastrointestinal pathogens simultaneously and have high sensitivity and specificity for several pathogens, including cryptosporidiosis (*8*,*9*).

This disease is monitored by the NYC Department of Health and Mental Hygiene (DOHMH) in part because NYC has a largely unfiltered water supply (*10*,*11*). Filtration is often used to facilitate *Cryptosporidium* oocyst removal in municipal water systems because the oocysts are resistant to standard concentrations of chemical disinfecting agents such as chlorine (*12*,*13*). Ultraviolet disinfection is now in place for the entire NYC water system, which can reduce the risk for cryptosporidiosis (*14*). Despite intensive surveillance, no evidence of drinking water–related outbreaks has been detected in NYC (*11*).

Although documented risks for cryptosporidiosis specifically in NYC include child care attendance (*15*) and HIV positivity (*16*), a systematic summary of the epidemiology of cryptosporidiosis in this large, urban setting is lacking. We examined all reported cryptosporidiosis cases since the first full year of routine investigations began in NYC in 1995. Trends in incidence, patient characteristics, and potential exposures in a large, urban setting are described, focusing on both the overall trend in annual incidence, as well as incidence trends relative to 2 major milestones in cryptosporidiosis surveillance: the period after widespread uptake of highly active antiretroviral therapy (HAART) for the control of AIDS (2000–2014), and the introduction of the sensitive syndromic multiplex panels for diagnosis (2015–2018).

## Methods

A case of cryptosporidiosis was included if it occurred in a person who resided in NYC at the time of diagnosis, was diagnosed during 1995–2018, was reported to the NYC DOHMH, and met the Council of State and Territorial Epidemiologists confirmed or probable case definition (*17*). Cases diagnosed by using a syndromic multiplex diagnostic were classified as confirmed by the 2012 Centers for Disease Control and Prevention/Council of State and Territorial Epidemiologists case definition (*18*). Over the study period, there were 5 separate case definitions. However, these modifications centered on classifying cases as probable versus confirmed and did not substantially affect the number of reported cases in NYC. NYC DOHMH epidemiologists attempted to interview all patients reported with cryptosporidiosis by telephone within several weeks of diagnosis. If a patient could not be located, a chart review or interview with a healthcare provider was performed. Information on diagnostic test type was available for each patient beginning in August 2012.

During an interview, we collected information on patient symptoms; demographic characteristics; and potential risk factors, such as travel, child care attendance, recreational water exposure, sexual activity (for patients >18 years of age), food and water consumption history, and HIV/AIDS diagnosis. All exposure information refers to reported exposures that occurred during the period of interest before symptom onset (defined as 1 month for cryptosporidiosis patients given a diagnosis during January 1995–April 2010 or 2 weeks for cryptosporidiosis patients given a diagnosis during and May 2010–December 2018).

### Population Data

We linearly interpolated intercensal population estimates for 1995–1999 by using 1990 and 2000 US Census data. Annual citywide, borough-specific, and demographic-specific intercensal population estimates (age group, sex, age/sex group, and race/ethnicity) for 2000–2018 were developed by the NYC DOHMH on the basis of the US Census Bureau’s Population Estimates Program, as of November 2019 (*19**–**21*).

To calculate incidence among persons immunocompromised because of HIV/AIDS, we used the number of cases among persons with HIV/AIDS (PLWHA) divided by the total annual population of PLWHA in NYC as determined by the HIV Epidemiology and Field Services Program, NYC DOHMH (*22*,*23*). The number of persons who did not have HIV/AIDS was estimated annually to be the total NYC population minus the population of PLWHA.

### Neighborhood-Level Poverty

Neighborhood-level poverty was defined as the percentage of census tract residents with household incomes <100% of the federal poverty level, according to data from the US Census and the American Community Survey (*24*,*25*). Census tract boundaries of the 2000 Census were used for patients given a diagnosis of cryptosporidiosis during 2000–2004, and census tract boundaries of the 2010 Census were used for patients given a diagnosis of cryptosporidiosis during 2005–2018. If a patient was homeless at the time of diagnosis, they were considered to live in very high poverty. Patients incarcerated at the time of diagnosis were excluded from poverty assignment. Population denominator data for neighborhood poverty for 2000–2018 were available from the NYC DOHMH Bureau of Epidemiology Services. These data are based on the American Community Survey; a 5-year pooled population estimate was used, centering on the year of the numerator, or the 5-year period that overlapped most closely in time with the year of the numerator (Table 1).

**Table 1:** Descriptive epidemiology of cryptosporidiosis in New York City, New York, USA, 2000–2014 and 2015*–*2018*

Category	2000–2014				2015–2018		
	No. cases	Incidence	IRR (95% CI)		No. cases	Incidence	IRR (95% CI)
No. cases	3,244	1.46			740	2.11	
Age group, y†							
<5	261	2.30	1.05 (0.83–1.32)		78	3.13	1.11 (0.76–1.60)
5–9	149	1.26	0.65 (0.50–0.85)		34	1.75	0.55 (0.35–0.86)
10–19	170	0.75	0.37 (0.29–0.48)		57	1.44	0.49 (0.33–0.73)
20–44	1,827	2.17	Referent		413	3.13	Referent
45–59	580	1.41	0.70 (0.57–0.86)		107	1.70	0.52 (0.36–0.74)
>59	254	0.39	0.22 (0.17–0.29)		51	0.69	0.24 (0.16–0.36)
Sex							
M	2,230	2.00	2.06 (1.70–2.49)		484	2.71	1.82 (1.34–2.48)
F	1,014	0.80	Referent		255	1.60	Referent
Sex and age group, y†							
All <20	580	1.26	0.43 (0.35–0.52)		169	2.19	0.57 (0.39–0.84)
Men 20–59	1,789	2.96	Referent		355	3.55	Referent
Men >59	135	0.60	0.22 (0.16–0.30)		33	1.06	0.30 (0.19–0.50)
Women >20	737	0.74	0.24 (0.20–0.30)		182	1.43	0.34 (0.23–0.51)
Borough of residence							
Bronx	583	1.59	0.57 (0.48–0.68)		129	2.09	0.54 (0.38–0.75)
Brooklyn	642	1.11	0.34 (0.28–0.40)		186	1.48	0.39 (0.28–0.54)
Manhattan	1,580	3.18	Referent		314	4.91	Referent
Queens	380	0.60	0.24 (0.19–0.29)		103	1.05	0.29 (0.21–0.41)
Staten Island	51	0.46	0.55 (0.38–0.80)		8	0.43	0.30 (0.13–0.66)
Race/ethnicity‡							
Hispanic	969	1.45	1.16 (0.96–1.40)		194	1.96	0.76 (0.56–1.04)
Non-Hispanic white	1,067	1.18	Referent		310	2.47	Referent
Non-Hispanic black/African American	855	1.77	1.45 (1.20–1.75)		122	1.54	0.65 (0.46–0.91)
Other	161	0.68	0.79 (0.61–1.03)		77	1.39	0.69 (0.47–1.01)
Immune status†‡							
Non-HIV/AIDS	1,349	0.65	Referent		544	1.56	Referent
HIV/AIDS	1,863	55.6	89.0 (70.2–112.3)		190	39.0	23.30 (16.4–33.2)
Neighborhood poverty‡§							
Low	883	1.18	Referent		203	2.10	Referent
Medium	769	1.56	1.03 (0.86–1.25)		197	1.97	0.86 (0.61–1.17)
High	583	1.46	1.23 (1.01–1.49)		151	2.16	0.97 (0.69–1.35)
Very high	917	1.87	1.42 (1.18–1.70)		188	2.45	1.17 (0.85–1.62)

*Incidence is median no. cases/100,000 persons; ACS, American Community Survey; IRR, incidence rate ratio. †Incidence and IRR not age-adjusted. ‡All categories had <10 missing values with the exception of race/ethnicity during 2000─2014 (n = 192) and 2015─2018 (n = 37), immune status during 2000─2014 (n = 32), and poverty during 2000─2014 (n = 92). §Classified by percent of census tract residents living below the federal poverty line (low, <10%; medium, 10%–<20%; high, 20%–<30%; very high, >30%); population denominator data were as follows for each listed year: 2000─2004 (2000 US Census); 2005─2009 (ACS 2007–2011); 2010 (2010 Census); 2011 (ACS 2009─2013); 2012 (ACS 2010─2014); 2013 (ACS 2011─2015); 2014─2018 (ACS 2012─2016).

### Demographic and Exposure Comparisons

We compared demographic characteristics for patients who were and were not able to be interviewed by using the χ^2^ test. In addition, we compared across age/sex groups the proportion of reported exposures during the patients’ incubation period among interviewed patients given a diagnosis during 2000–2018 by using the χ^2^ test.

### Age Adjustment and Incidence Calculations

Annual incidence during 2000–2018 was age-adjusted by using direct standardization for age at diagnosis and weighting by the US 2000 standard population (*26*). We calculated age-adjusted annual disease rates for the period 1995–2018 for the city and by borough of residence. We also calculated age-adjusted disease rates by demographic strata for 2 periods: the period after widespread adoption of HAART and before the introduction of syndromic multiplex panels (2000–2014) and the period after the introduction of syndromic multiplex panels (2015–2018). The demographic characteristics of interest included age, sex, age/sex group (persons of all sexes <20 years of age, men 20–59 years of age, men >59 years of age, and women >20 years of age), borough of residence, race/ethnicity, and neighborhood poverty level. We also calculated unadjusted cryptosporidiosis rates for persons with and without HIV/AIDS. We provide median annual incidence for the entire surveillance period (1995–2018) and for 2000–2014 and 2015–2018.

We calculated age-adjusted incidence rate ratios (IRRs) with 95% CIs by using negative binomial regression, in which the outcome was annual case count, the exposure of interest was a demographic characteristic (e.g., sex), age group was included as a potential confounder, and the offset term was the log of the population per year. We conducted 2 sets of analyses: the first compared rates among strata of demographic groups in the 2 different periods and the second compared rates of each demographic strata between the 2 periods. We conducted all analyses by using SAS version 9.4 (SAS Institute, https://www.sas.com).

### Mapping

We mapped incidence of cryptosporidiosis at the community district (CD) level (n = 59). Annual CD population estimates for 2000–2018 were produced by DOHMH on the basis of the US Census Bureau Population Estimates Program, as of November 2019. Median annual incidence was mapped by CD over time and for specific subsets of age and sex. Age-adjusted incidence rates were mapped by using equal interval breaks. Almost all (98%, 3,894/3,984) patients had a successfully geocoded address.

## Results

### Trends in Cryptosporidiosis Incidence during 1995–2018

During 1995–2018, a total of 3,984 cryptosporidiosis cases were diagnosed and reported to the NYC DOHMH. Interviews were completed for 3,295 (83%) patients. The most common reasons for failure to interview were because the NYC DOHMH was unable to locate the patient (7%), the patient died (4%), or the patient refused to be interviewed (3%). Because death certificate information was not available, cause of death could not be determined. Several major differences were identified between patients who were and were not interviewed (Appendix Table 1). Patients who were not interviewed during 1995–2018 were more likely to be men 20–59 years of age, to be non-Hispanic black/African American, to have a known diagnosis of HIV/AIDS, and to live in census tracts with high or very high poverty levels. A total of 13 patients were incarcerated and 10 patients were homeless at the time of diagnosis.

The median annual age-adjusted incidence of reported cryptosporidiosis cases in NYC during 1995–2018 was 1.71 cases/100,000 persons (interquartile range 1.31–2.18 cases/100,000 persons). The citywide annual age-adjusted incidence decreased by 65% from 1995 (5.86 cases/100,000 persons) through 2000 (2.07 cases/100,000 persons) (Figure 1, panel A). The rate increased toward the end of the study period from 1.59 cases/100,000 persons in 2015 to 2.99 cases/100,000 persons in 2018. Cryptosporidiosis incidence was highest in Manhattan throughout the study period (Figure 1, panel B). Age-adjusted borough-specific annual incidence decreased in Manhattan most dramatically over time from 15.27 cases/100,000 persons in 1995 to 5.12 cases/100,000 persons in 2000. Rates remained relatively stable in each borough during 2000–2014 and began to increase in Brooklyn, the Bronx, and Queens after 2015 (Figure 1, panel B).

**Figure 1:** Age-adjusted annual incidence of cryptosporidiosis/100,000 persons, New York City, New York, USA, 1995–2018. A) Citywide; B) by borough of residence.

### Trends in Cryptosporidiosis Incidence and Epidemiology during 2000–2018

A total of 2,539 cryptosporidiosis patients were given a diagnosis during 2000–2018 and reported to the NYC DOHMH. Of the 5 boroughs of NYC, cryptosporidiosis patients most often resided in Manhattan (44%), followed by Brooklyn (24%) and the Bronx (19%). Most (54%) patients were men 20–59 years of age; patients were most also frequently non-Hispanic white (33%), followed by Hispanic (any race) (28%) and non-Hispanic black/African American (26%) (Appendix Table 2). The median annual citywide age-adjusted incidence increased from 1.46 cases/100,000 persons during 2000–2014 to 2.11 cases/100,000 persons during 2015–2018. Age group–specific incidence was highest among those <5 years of age, followed by those 20–44 years of age during both periods (Table 1). The proportion of cryptosporidiosis patients with a known diagnosis of HIV/AIDS decreased over time from 60% during 2000–2004 to 26% during 2015–2018 (Appendix Table 2); the incidence was higher among PLWHA throughout 2000–2018 (Table 1).

### Cryptosporidiosis among Men during 2000–2018

Incidence among men was higher throughout 2000–2018, and incidence for those 20–59 years of age specifically was consistently higher than for all other age/sex groups during 2000–2014 (median 2.96 cases/100,000 persons) and 2015–2018 (median 3.55 cases/100,000 persons) (Table 1; Figure 2). We identified exposures during the incubation period reported by cryptosporidiosis patients with a completed interview by age and sex group (Table 2). Men 20–59 years of age were more likely to report high-risk sexual practices with an increased risk for fecal contact (43%) compared with older men (16%) and women (17%). In addition, 2 neighborhoods had consistently increased age-adjusted incidence rates before introduction of syndromic multiplex diagnostics; including Chelsea/Greenwich Village and Inwood/Washington Heights in Manhattan (Figure 3, panel A). The geography of age-adjusted incidence among men 20–59 years of age (Figure 3, panel C) differed substantially from other patients (Figure 3, panel D) in that cryptosporidiosis patients who were men were concentrated again specifically in the Chelsea/Greenwich Village neighborhoods.

**Figure 2:** Annual cryptosporidiosis incidence (cases/100,000 persons) by sex and age group, New York City, New York, USA, 2000–2018. A) Male. B) Female.

**Table 2:** Reported exposures of cryptosporidiosis patients stratified by those with and without international or domestic travel outside New York City, New York, USA, during period of interest before symptom onset for patients, 2000*–*2018*

Characteristic	Age, y/sex group, no. positive/no. tested (%)					p value
	<20/All	20–59/M	>59/M	>20/F	Total	
Interviewed patients	500/555 (90.1)	1,000/1,359 (73.6)	71/87 (81.6)	434/537 (80.8)	2,005/2,538 (79)	<0.001
International travel	254/492 (51.6)	158/993 (15.9)	16/72 (22.2)	155/431 (36.0)	583/1,988 (29.3)	<0.001
Domestic travel	99/445 (22.2)	208/910 (22.9)	12/65 (18.5)	81/395 (20.5)	400/1,815 (22.0)	0.77
No travel	149/474 (31.4)	562/908 (61.9)	39/67 (58.2)	179/401 (44.6)	929/1,850 (50.2)	<0.001
High-risk sex†	ND	234/546 (42.9)	6/37 (16.2)	30/174 (17.2)	270/757 (35.7)	<0.001
Recreational water contact‡	35 (23.5)	46 (8.2)	3 (7.7)	12 (6.7)	96 (10.3)	<0.001
Child care attendee§	14/61 (23.0)	ND	ND	ND	ND	NA
Animal contact¶	36 (24.2)	197 (35.1)	5 (12.8)	64 (35.8)	302 (32.5)	0.02
Ate high-risk food#	61 (40.9)	338 (60.2)	16 (41.0)	113 (63.1)	528 (56.9)	<0.001
Drank NYC tap water	68 (45.6)	403 (71.7)	23 (59.0)	119 (66.5)	613 (66.0)	<0.001
Treated NYC tap water**	53 (35.8)	217 (38.6)	16 (41.0)	73 (40.8)	359 (38.7)	0.50
Used NYC tap water††	117 (78.5)	496 (88.3)	31 (79.5)	165 (92.2)	809 (87.1)	<0.001
Drank high-risk water‡‡	2/145 (1.4)	8/549 (1.5)	0/37 (0)	2/174 (1.1)	12/905 (1.3)	>0.99

*Percentages in parentheses reflect percentage of patients who were interviewed during each period. Period of interest is defined as 1 month for patients given a diagnosis during January 1995–April 2010, and 2 weeks for patients given a diagnosis during May 2010–December 2018. NA, not applicable; ND, no data were collected; NYC, New York City. †For adults >18 years of age, defined as having anal sex (receptive or insertive), insertion of a finger or a tongue in or around a partner’s anus, or having oral sex. ‡Defined as water from a hot tub, public or private swimming pool, stream, ocean, or recreational water park. §For children <5 years of age. ¶Defined as having a pet, being exposed to an animal, changing cat litter, picking up dog stool, visiting a zoo, having a pet, or having a job that required contact with animals. #Defined as raw or unpasteurized cheese, milk or cider, cooked or uncooked shellfish, and unpeeled fruit or vegetables. **Patients either boiled or filtered NYC municipal water before drinking. ††Used NYC municipal water to brush teeth, wash vegetables/fruit, make ice, or make juice. ‡‡High-risk water was defined as water from a stream, spring, pond, or private well.

**Figure 3:** Median annual cryptosporidiosis incidence (cases/100,000 persons) by community district (CD), New York City New York, USA, 1995–2018. A) All persons, age-adjusted, 2000–2014, showing CDs that include Chelsea (Chelsea, Clinton, Hudson Yards) and Greenwich Village (Greenwich Village, Hudson Square, Little Italy, NoHo, SoHo, South Village, West Village). B) All persons, age-adjusted, 2015–2018. C) Men 20–59 years of age, 2015–2018. D) All persons <20 years of age and men >59 years of age, 2015–2018.

### International Travel and NYC Tap Water Exposure during 2000–2018

Approximately 30% of patients given a diagnosis during 2000–2018 reported international travel during their incubation period (Table 2). Patients given a diagnosis of cryptosporidiosis during 2015–2018 were more likely to report international travel (34%) compared with patients given a diagnosis during 2000–2014 (23%) (p<0.0001). Compared with patients >20 years of age (52%), patients <20 years of age (22%) were more likely to have traveled internationally. The most common international destinations were the Dominican Republic (23% of all international destinations) and Mexico (12% of all international destinations). More than two thirds of patients who reported traveling to the Dominican Republic were <20 years of age (68%, 96/142); a similar pattern was not identified for patients with reported travel to Mexico. Finally, patients traveling to the Dominican Republic most often lived in Inwood/Washington Heights in NYC (28%, 39/140).

In terms of NYC municipal water use, most adults reported drinking plain tap water, and >75% of all patients reported using plain tap water for daily activities during their incubation periods. Furthermore, among patients with no reported travel outside NYC, patients <20 years of age were most likely to report recreational water contact (24%).

### Impact of Syndromic Multiplex Panels

The median age-adjusted annual incidence increased after introduction of syndromic multiplex panels (IRR for 2015–2018 vs. 2000–2014 1.49, 95% CI 1.17–1.91). Incidence increased during 2015–2018 among most examined demographic groups, most dramatically among those without a known diagnosis of HIV/AIDS (IRR 2.37, 95% CI 1.75–3.22), those 10–19 years of age (IRR 2.07, 95% CI 1.35–3.19), and those who were non-Hispanic white (IRR 1.91, 95% CI 1.46–2.50) (Appendix Table 3). Compared with non-Hispanic whites, the median age-adjusted incidence among non-Hispanic black/African Americans (1.77 cases/100,000 persons) was higher before introduction of syndromic multiplex panels (IRR 1.45, 95% CI 1.20–1.75). However, after introduction of these diagnostics, the incidence in non-Hispanic whites (2.47 cases/100,000 persons) was higher than that for all other race/ethnicity groups (Table 1).

The introduction of syndromic multiplex panels led to an increase in detection across a broader number of neighborhoods across Manhattan (Figure 3, panel B). Before introduction of syndromic multiplex panels, the incidence of cryptosporidiosis was higher among neighborhoods of very high poverty (1.87 cases/100,000 persons) compared with low poverty (IRR 1.42, 95% CI 1.18–1.70). This association is probably driven by an increased number of cases among very high poverty neighborhoods during 2004 (cause unknown). During 2015–2018, neighborhood poverty was not associated with cryptosporidiosis incidence.

Before the introduction of syndromic multiplex panels, cryptosporidiosis diagnoses increased during July–October annually, peaking in August (median 45 cases, range 8–30 cases) and September (median 17 cases, range 7–35 cases) (Figure 4, panel A). After introduction of these diagnostics, the monthly case counts increased substantially in the same months of August (median 30 cases, range 13–35 cases) and September (median 35 cases, range 28–39 cases) (Figure 4, panel B). Patients <20 years of age were most likely to have diagnosis dates in the late summer both before (August: 23%, September 28%) (Figure 4, panel C) and after (August 23%, September 27%) (Figure 4, panel D) introduction of syndromic multiplex panels.

**Figure 4:** Seasonality of cryptosporidiosis in New York City, New York, USA, 1995–2018. A, B) Count of cryptosporidiosis cases by month during 2000–2014 (A) and 2015–2018 (B). Horizontal bars within boxes indicate median case count by month; box bottoms and tops indicate 25th and 75th percentiles; dots indicate outliers (>95th percentile); and error bars indicate 95% CIs. C, D) Percentage of patients by month of diagnosis and age group during 2000–2014 (C) and 2015–2018 (D).

During August 2012–December 2018, a total of 943 patients received a diagnosis of cryptosporidiosis by 992 primary tests at a commercial or hospital laboratory. Before mid-2015, most (76%) patients had only 1 type of positive test result, which consisted of either microscopy or ELISA for *Cryptosporidium* antigen. However, after introduction of syndromic multiplex panels, the proportion of all patients who were given a diagnosis of cryptosporidiosis by these panels increased from 18% (25/137) during 2015 to 76% (190/250) during 2018 (Figure 5). More than 97% (345/355) of patients whose stool specimens were tested by using a syndromic multiplex panel during 2015–2018 were exclusively given a diagnosis by use of this assay rather than through an additional test by microscopy or an antigen ELISA. Most (74%, 255/345) cryptosporidiosis patients given a diagnosis by use of the syndromic multiplex panel did not have another enteric co-infection.

**Figure 5:** Count of positive diagnostic tests for cryptosporidiosis by month, New York City, New York, USA, August 2012–December 2018. Diagnostic tests include microscopy (stain or ova and parasite test), antigen ELISA for *Cryptosporidium* antigen, and syndromic multiplex test. A patient can have >1 diagnostic test/disease episode.

## Discussion

An analysis of cryptosporidiosis surveillance data collected in NYC during 1995–2018 suggests that the epidemiology of the parasitic enteric infection in this large, urban, international setting is multifaceted. Although the incidence of cryptosporidiosis in NYC decreased dramatically after the introduction of HAART in the mid-1990s, incidence remained consistently higher among men in NYC, probably reflecting an enduring burden of disease in men who have sex with men (MSM). We also found that cryptosporidiosis was common in children in NYC, probably driven by summertime international travel to disease-endemic areas. Finally, the recent introduction of syndromic multiplex diagnostic panels has led to not only a major increase in the reported incidence of cryptosporidiosis but also to a shift in the demographic makeup of reported cryptosporidiosis patients in NYC.

Our data show that the burden of cryptosporidiosis has been consistently elevated among adult men 20–59 years of age in NYC, probably driven by person-to-person sexual transmission among MSM. Although we did not have reliable data on patient MSM status, high rates of cryptosporidiosis among men were consistently identified in areas known to have an above-average proportion of residents who are MSM, such as Chelsea (*27*). Patients in this age/sex group were more likely to report sexual practices with increased risk for fecal contact during the incubation period than were those in any other age/sex group in NYC. MSM are historically at greater risk for cryptosporidiosis, not only because of a higher prevalence of AIDS in this population (*28*) but also because of sexual practices, such as anilingus, that entail a low risk for HIV transmission but increase the risk for fecal contact (*3*).

The dramatic decrease in citywide cryptosporidiosis incidence observed during 1995–2000 is consistent with the uptake of HAART among PLWHA in NYC. Cryptosporidiosis is a well-known opportunistic infection among PLWHA (*1*,*29*), and restoration of immune function is the recommended therapy (*30*). The number of deaths from all causes among persons who have AIDS peaked in 1994 in NYC and decreased substantially after the introduction of HAART (*31*). Although cryptosporidiosis rates remain high for PLWHA in NYC, patients without HIV/AIDS now represent most reported patients. This finding is likely not only because of high HAART coverage among patients with HIV/AIDS (*32*) but also because of increased case finding in the general population after introduction of syndromic multiplex diagnostics. Before introduction of these new diagnostics, physicians needed to specifically request cryptosporidiosis testing as part of the traditional ova and parasite testing. Given the dramatic consequences of cryptosporidiosis among PLWHA, clinicians treating cryptosporidiosis in this population would have likely been more aware of the need to specifically request testing for this parasite before presyndromic multiplex panels were introduced.

In addition to patients who were HIV negative, the introduction of syndromic multiplex testing led to an increase in reported cases among persons who were non-Hispanic white. This finding might reflect the populations residing in the specific catchment areas of the laboratories that use syndromic multiplex panels, as well as disparities in healthcare access by race/ethnicity (*33*). We also identified a higher and increasing burden of cryptosporidiosis among children in NYC, especially after introduction of syndromic multiplex panels. Because children with diarrhea are more likely to be taken to a healthcare facility than adults (*34*), the increasing incidence of cryptosporidiosis in the younger age group probably reflects healthcare-seeking behavior along with the improved diagnostic sensitivity of syndromic multiplex panels.

Our data also show that children were more likely to be given a diagnosis of cryptosporidiosis during the summer months and were most likely to report recreational water contact and international travel, particularly to the Dominican Republic, where they might be in contact with contaminated food or water (*35*). Although recreational water contact is a known risk factor for cryptosporidiosis (*2*), no large pool-related outbreaks have been detected in NYC. In addition, most cryptosporidiosis patients reported drinking NYC municipal tap water and using it for household needs. Extensive surveillance is in place to monitor for citywide waterborne cryptosporidiosis and giardiasis outbreaks (*11*). To date, there have been no outbreaks of waterborne disease related to the NYC municipal tap water.

Our study has several limitations. Interviewed patients tended to be of higher socioeconomic status than those who were not interviewed, which might bias our results because wealthier patients might have different risk exposures than patients who live in neighborhoods of high poverty. Furthermore, the period of interest for risk factor exposure decreased during 2010, which might have reduced the proportion of persons reporting risk factors over time. Finally, some race and ethnicity data were not collected by self-report and therefore might not accurately capture self-identified race and ethnicity of patients. Notwithstanding these limitations, the epidemiology of cryptosporidiosis merits continued attention because the incidence will probably continue to increase in NYC, as well as in other jurisdictions across the United States (*7*), given the cost-effectiveness of syndromic multiplex panels (*36*). Public health surveillance activities will need to take the improved case detection and increases in hospital/laboratory-specific catchment areas into account when guiding public health action (*37*).

In conclusion, the epidemiology of cryptosporidiosis in NYC reflects the diverse population of this city. The consistently elevated burden in men probably related to sexual transmission among MSM warrants outreach to this community and their providers to increase awareness. Furthermore, messaging related to prevention and treatment for cryptosporidiosis should be targeted to international travelers, particularly parents of children in NYC who travel to the Dominican Republic during the summer months. We anticipate the reported incidence of cryptosporidiosis to continue to increase with the increasing use of syndromic multiplex panels and that the demographic profiles of the patients might also change as these new diagnostics are adopted more widely. These observed patterns of disease might also be present in other urban, diverse jurisdictions, both nationally and internationally.

Appendix Additional information on epidemiology of cryptosporidiosis, New York City, New York, USA, 1995–2018.

## References

[1] Chen XM, Keithly JS, Paya CV, LaRusso NF. Cryptosporidiosis. N Engl J Med. 2002;346:1723–31. 10.1056/NEJMra01317012037153

[2] Yoder JS, Beach MJ. *Cryptosporidium* surveillance and risk factors in the United States. Exp Parasitol. 2010;124:31–9. 10.1016/j.exppara.2009.09.02019786022

[3] Hellard M, Hocking J, Willis J, Dore G, Fairley C. Risk factors leading to *Cryptosporidium* infection in men who have sex with men. Sex Transm Infect. 2003;79:412–4. 10.1136/sti.79.5.41214573839PMC1744752

[4] Roy SL, DeLong SM, Stenzel SA, Shiferaw B, Roberts JM, Khalakdina A, et al.; Emerging Infections Program FoodNet Working Group. Risk factors for sporadic cryptosporidiosis among immunocompetent persons in the United States from 1999 to 2001. J Clin Microbiol. 2004;42:2944–51. 10.1128/JCM.42.7.2944-2951.200415243043PMC446318

[5] Checkley W, White AC Jr, Jaganath D, Arrowood MJ, Chalmers RM, Chen X-M, et al. A review of the global burden, novel diagnostics, therapeutics, and vaccine targets for *cryptosporidium.* Lancet Infect Dis. 2015;15:85–94. 10.1016/S1473-3099(14)70772-825278220PMC4401121

[6] New York City Health Code. Article 11: reportable diseases and conditions; 2019 [cited 2019 Dec 5]. ttps://www1.nyc.gov/assets/doh/downloads/pdf/about/healthcode/health-code-article11.pdf

[7] Marder EP, Cieslak PR, Cronquist AB, Dunn J, Lathrop S, Rabatsky-Ehr T, et al. Incidence and trends of infections with pathogens transmitted commonly through food and the effect of increasing use of culture-independent diagnostic tests on surveillance: Foodborne Diseases Active Surveillance Network, 10 U.S. Sites, 2013–2016. MMWR Morb Mortal Wkly Rep. 2017;66:397–403. 10.15585/mmwr.mm6615a128426643PMC5687182

[8] Buss SN, Leber A, Chapin K, Fey PD, Bankowski MJ, Jones MK, et al. Multicenter evaluation of the BioFire FilmArray gastrointestinal panel for etiologic diagnosis of infectious gastroenteritis. J Clin Microbiol. 2015;53:915–25. 10.1128/JCM.02674-1425588652PMC4390666

[9] Navidad JF, Griswold DJ, Gradus MS, Bhattacharyya S. Evaluation of Luminex xTAG gastrointestinal pathogen analyte-specific reagents for high-throughput, simultaneous detection of bacteria, viruses, and parasites of clinical and public health importance. J Clin Microbiol. 2013;51:3018–24. 10.1128/JCM.00896-1323850948PMC3754681

[10] New York City Department of Environmental Protection. Filtration avoidance determination (FAD) Reports; 2018 [cited 2019 Dec 5]. http://www.nyc.gov/html/dep/html/watershed_protection/fad.shtml

[11] Alleyne L, Thompson C, Fitzhenry R, Seeley A, Mathes R, Janus L. Waterborne disease risk assessment program 2018 annual report; 2019 [cited 2019 Dec 5]. https://www1.nyc.gov/html/dep/pdf/reports/fad-8.1-waterborne-disease-risk-assessment-program-annual-report-03-19.pdf

[12] United States Environmental Protection Agency. Interim enhanced surface water treatment rule; December 1998 [cited 2019 Dec 5]. https://www.epa.gov/dwreginfo/surface-water-treatment-rules

[13] Meinhardt PL, Casemore DP, Miller KB. Epidemiologic aspects of human cryptosporidiosis and the role of waterborne transmission. Epidemiol Rev. 1996;18:118–36. 10.1093/oxfordjournals.epirev.a0179209021307

[14] Morita S, Namikoshi A, Hirata T, Oguma K, Katayama H, Ohgaki S, et al. Efficacy of UV irradiation in inactivating *Cryptosporidium parvum* oocysts. Appl Environ Microbiol. 2002;68:5387–93. 10.1128/AEM.68.11.5387-5393.200212406729PMC129916

[15] Crawford FG, Vermund SH, Ma JY, Deckelbaum RJ. Asymptomatic cryptosporidiosis in a New York City day care center. Pediatr Infect Dis J. 1988;7:806–7. 10.1097/00006454-198811000-000133231502

[16] Spencer KL, Soave R, Acosta A, Gellin B, Prince A, Ramos L, et al. Cryptosporidiosis in HIV-infected persons: prevalence in a New York City population. Int J Infect Dis. 1997;1:217–21. 10.1016/S1201-9712(97)90041-2

[17] Council of State and Territorial Epidemiologists. Cryptosporidiosis (*Cryptosporidium* spp.) case definition. Position statement; 2012 [cited 2019 Dec 5]. https://wwwn.cdc.gov/nndss/conditions/cryptosporidiosis/case-definition/2012

[18] Centers for Disease Control and Prevention. Cryptosporidiosis (*Cryptosporidium* spp.): case definition(s). National Notifiable Diseases Surveillance System; 2017 [cited 2019 Dec 5]. https://wwwn.cdc.gov/nndss/conditions/cryptosporidiosis

[19] New York City Department of City Planning. Decennial census; 2010 [cited 2019 Dec 5]. https://www1.nyc.gov/site/planning/data-maps/nyc-population/census-2010.page

[20] New York City Department of City Planning. Decennial census; 2000 [cited 2019 Dec 5]. http://www1.nyc.gov/site/planning/data-maps/nyc-population/census-2000.page

[21] New York City Department Of Health and Mental Hygiene. EpiQuery census 1990–2000 and 2010 modules; 2018 [cited 2019 Dec 5]. https://www1.nyc.gov/site/doh/data/data-sets/epi-census.page

[22] New York City Department of Health and Mental Hygiene HIV Epidemiology and Field Services Program. AIDS diagnosis and persons living with HIV/AIDS by year, Pre-1981 to 2016, New York City; 2016 [cited 2019 Dec 5]. https://www1.nyc.gov/assets/doh/downloads/pdf/ah/surveillance-trend-tables.pdf

[23] New York City Department of Health and Mental Hygiene. HIV surveillance annual report 2017; 2018 [cited 2019 Dec 5]. https://www1.nyc.gov/assets/doh/downloads/pdf/dires/hiv-surveillance-annualreport-2017.pdf

[24] US Census Bureau. American community survey (ACS); 2016 [cited 2019 Dec 5]. https://census.gov/programs-surveys/acs/data.html

[25] Toprani A, Hadler J. Selecting and applying a standard area-based socioeconomic status measure for public health data: analysis for New York City. New York City Department of Health and Mental Hygiene: Epi Research Report; May 2013 [cited 2019 Dec 5]. https://www1.nyc.gov/assets/doh/downloads/pdf/epi/epiresearch-SES-measure.pdf

[26] Klein RJ, Schoenborn CA. Age adjustment using the 2000 projected US population. Hyattsville (MD): Centers for Disease Control and Prevention, National Center for Health Statistics; 2001.11676466

[27] Bureau of Epidemiology Services New York City Department of Health and Mental Hygiene. Prevalence of men Who had sex with men in the past 12 months in NYC by United Hospital Fund Neighborhood, community health survey, 2012–2016; 2017 [cited 2019 Dec 5]. http://www1.nyc.gov/site/doh/data/data-sets/community-health-survey-public-use-data.page

[28] Centers for Disease Control and Prevention (CDC). Epidemiology of HIV/AIDS—United States, 1981-2005. MMWR Morb Mortal Wkly Rep. 2006;55:589–92.16741494

[29] Hunter PR, Nichols G. Epidemiology and clinical features of *Cryptosporidium* infection in immunocompromised patients. Clin Microbiol Rev. 2002;15:145–54. 10.1128/CMR.15.1.145-154.200211781272PMC118064

[30] Sparks H, Nair G, Castellanos-Gonzalez A, White AC Jr. Treatment of *Cryptosporidium*: what we know, gaps, and the way forward. Curr Trop Med Rep. 2015;2:181–7. 10.1007/s40475-015-0056-926568906PMC4640180

[31] Nash D, Katyal M, Shah S. Trends in predictors of death due to HIV-related causes among persons living with AIDS in New York City: 1993-2001. J Urban Health. 2005;82:584–600. 10.1093/jurban/jti12316237203PMC3456686

[32] Sackoff JE, Hanna DB, Pfeiffer MR, Torian LV. Causes of death among persons with AIDS in the era of highly active antiretroviral therapy: New York City. Ann Intern Med. 2006;145:397–406. 10.7326/0003-4819-145-6-200609190-0000316983127

[33] Bailey ZD, Krieger N, Agénor M, Graves J, Linos N, Bassett MT. Structural racism and health inequities in the USA: evidence and interventions. Lancet. 2017;389:1453–63. 10.1016/S0140-6736(17)30569-X28402827

[34] Van Cauteren D, De Valk H, Vaux S, Le Strat Y, Vaillant V. Burden of acute gastroenteritis and healthcare-seeking behaviour in France: a population-based study. Epidemiol Infect. 2012;140:697–705. 10.1017/S095026881100099921676346

[35] Steffen R, Hill DR, DuPont HL. Traveler’s diarrhea: a clinical review. JAMA. 2015;313:71–80. 10.1001/jama.2014.1700625562268

[36] Goldenberg SD, Bacelar M, Brazier P, Bisnauthsing K, Edgeworth JD. A cost benefit analysis of the Luminex xTAG Gastrointestinal Pathogen Panel for detection of infectious gastroenteritis in hospitalised patients. J Infect. 2015;70:504–11. 10.1016/j.jinf.2014.11.00925449904

[37] Peterson ER, Fireteanu AM, Greene SK. Adapting reportable disease cluster detection. Methods for increased use of culture-independent diagnostic testing. Presented at: Annual Meeting of the Council of State and Territorial Epidemiologists; 2018 Jun 11–13; West Palm Beach, Florida, USA [cited 2019 Dec 5]. https://cste.confex.com/cste/2018/meetingapp.cgi/Paper/9135

# Review article

## Earning CME Credit

To obtain credit, you should first read the journal article. After reading the article, you should be able to answer the following, related, multiple-choice questions. To complete the questions (with a minimum 75% passing score) and earn continuing medical education (CME) credit, please go to http://www.medscape.org/journal/eid. Credit cannot be obtained for tests completed on paper, although you may use the worksheet below to keep a record of your answers. 

You must be a registered user on http://www.medscape.org. If you are not registered on http://www.medscape.org, please click on the “Register” link on the right hand side of the website. 

Only one answer is correct for each question. Once you successfully answer all post-test questions, you will be able to view and/or print your certificate. For questions regarding this activity, contact the accredited provider, CME@medscape.net. For technical assistance, contact CME@medscape.net. American Medical Association’s Physician’s Recognition Award (AMA PRA) credits are accepted in the US as evidence of participation in CME activities. For further information on this award, please go to https://www.ama-assn.org. The AMA has determined that physicians not licensed in the US who participate in this CME activity are eligible for *AMA PRA* *Category 1 Credits™*. Through agreements that the AMA has made with agencies in some countries, AMA PRA credit may be acceptable as evidence of participation in CME activities. If you are not licensed in the US, please complete the questions online, print the AMA PRA CME credit certificate, and present it to your national medical association for review.

## Article Title:  Epidemiology of Cryptosporidiosis, New York City, New York, USA, 1995–2018

## CME Questions

1. **Which of the following statements regarding cryptosporidiosis is most accurate?**
  - It is usually transmitted through either the respiratory or fecal-oral route
  - Recreational water use is a risk factor for cryptosporidiosis
  - All patients with cryptosporidiosis require antiparasitic treatment
  - The best means to neutralize Cryptosporidium oocytes in drinking water is low levels of chlorine
2. **What does the current study by Alleyne and colleagues find regarding the epidemiology of cryptosporidiosis in New York, New York?**
  - The median annual age-adjusted incidence of cryptosporidiosis cases was 536/100,000 population
  - There was a gradual increase in the incidence of cryptosporidiosis from 1995 to 2018
  - Cryptosporidiosis was most active in January and February
  - There were no outbreaks of cryptosporidiosis related to New York, New York tap water
3. **Which of the following statements regarding individuals with documented cryptosporidiosis in the current study is most accurate?**
  - Most patients were male between the ages of 20 and 59 years
  - Most patients were nonwhite
  - The incidence of cryptosporidiosis among individuals living with HIV infection increased steadily from 1995 to 2018
  - There was no geographic pattern of cryptosporidiosis cases
4. **Which of the following statements regarding other trends in the epidemiology of cryptosporidiosis in the current study is most accurate?**
  - Most patients reported international travel during their incubation period
  - Cryptosporidiosis related to international travel declined in incidence between 2010 and 2018
  - Syndromic multiplex panels were associated with an increase in the incidence of cryptosporidiosis
  - The introduction of syndromic multiplex panels led to an increase in cases of cryptosporidiosis among individuals living with HIV infection
